# Correlation between maternal and fetal heart rate increases with fetal mouse age in typical development and is disturbed in autism mouse model treated with valproic acid

**DOI:** 10.3389/fpsyt.2022.998695

**Published:** 2022-11-24

**Authors:** Namareq Widatalla, Ahsan Khandoker, Chihiro Yoshida, Kana Nakanishi, Miyabi Fukase, Arisa Suzuki, Masatoshi Saito, Yoshitaka Kimura, Yoshiyuki Kasahara

**Affiliations:** ^1^Next Generation Biological Information Technology, Tohoku University Graduate School of Biomedical Engineering, Sendai, Japan; ^2^Healthcare Engineering Innovation Center, Department of Biomedical Engineering, Khalifa University, Abu Dhabi, United Arab Emirates; ^3^Department of Maternal and Fetal Therapeutics, Tohoku University Graduate School of Medicine, Sendai, Japan; ^4^Advanced Interdisciplinary Biomedical Engineering, Tohoku University Graduate School of Medicine, Sendai, Japan; ^5^Department of Maternal and Child Health Care Medical Science, Tohoku University Graduate School of Medicine, Sendai, Japan; ^6^Department of Obstetrics and Gynecology, Tohoku University Graduate School of Medicine, Sendai, Japan

**Keywords:** maternal–fetal RRI similarity, fetal mouse development, fetal programming, autonomic nervous system, autism spectrum disorder

## Abstract

**Introduction:**

Autism spectrum disorder (ASD) is considered a significant behavioral problem that is characterized by impairment in social interaction and communication. It is believed that some cases of ASD originate in the intrauterine maternal environment. Therefore, we hypothesized that there might be qualitative changes in the interaction between the mother and fetus in ASD during the prenatal period, hence, we investigated the similarity patterns between maternal and fetal heart rate (HR).

**Methods:**

In this study, we first demonstrate the presence and formation of similarities between maternal and fetal RR interval (RRI) collected from typical developmental mice at different embryonic days (EDs), ED13.5, ED15.5, ED17.5, and ED18.5. The similarities were quantified by means of cross-correlation (CC) and magnitude-squared coherence (MSC) analyses. Correlation analysis between the CC coefficients and EDs and between MSC coefficients and EDs showed that the same coefficients increase with EDs, suggesting that similarities between maternal and fetal RRI are associated with typical fetal development. Next, because maternal and fetal similarities were indicative of development, a comparison analysis between the autism mouse model (injected with valproic acid (VPA)), and the control group (injected with saline) was performed for ED15.5 and ED18.5.

**Results:**

The results of the comparison showed that the CC and MSC coefficients of VPA fetuses were significantly lower than that of the control group. The lower coefficients in VPA-treated mice suggest that they could be one of the features of ASD symptoms. The findings of this study can assist in identifying potential ASD causes during the prenatal period.

## Introduction

Autism spectrum disorder (ASD) is a neurodevelopmental disorder characterized by restrictive interest and impairment in social interaction and communication. ASD is considered a significant neurological disorder among children (1, 2). Compared to non-autistic children, children with autism face more challenges in learning and adapting to the surrounding environment due to their disturbed cognitive capabilities (2). So far, there is no medication to cure autism (1), but early diagnosis of the disorder may enhance a child's cognitive abilities through early treatment (3, 4). Early diagnosis of ASD is generally challenging because diagnosis is based on behavioral assessments rather than clinical tests (1). Hence, without behavioral markers, diagnosis of ASD is difficult or almost impossible. The onset of the disorder is variable (2) and on average, diagnosis of ASD is possible around 2.5–3 years (3, 4).

The etiology behind ASD is not fully understood yet and it is believed that different factors may lead to ASD (1). Maternal uptake of certain drugs, such as valproic acid (VPA), during pregnancy may increase the risk for Autism (1, 5). VPA is an antiepileptic drug and mood stabilizer, and previously, it was reported that fetuses who were exposed to it had higher risks of developing autism and cognitive disorders (6–8). The latter signifies the need to identify autistic features from as early as the prenatal period. Since the fetus is inaccessible, fetal development is broadly monitored by heart rate (HR) and HR variability (HRV) assessments (9, 10). HRV has been used as an index for autonomic nervous system (ANS) activity (10, 11) and previously, it was demonstrated that HRV was found to be reduced in patients with ASD compared to neurotypical subjects (12, 13). In our previous study (5), we developed an ASD model by injection of VPA into maternal mice on embryonic day 12.5 (ED12.5). We showed that HRV patterns of fetuses and offspring of mothers treated with VPA were different compared to control mice (injected with saline). Hence, based on the previously mentioned studies, changes manifested in fetal HR patterns could be a potential feature of ASD during the prenatal period.

Previously (5), we have not looked at maternal-fetal HR coupling but in our recent study (14), we calculated the average mean values of RR interval (RRI) from 1-min segments at ED15.5, and we found that a positive significant correlation existed between maternal and fetal RRI in the control group, on the other hand, the same correlation was not found in the VPA group (14). The differences in correlation that we found in our previous study (14) suggest the presence of communication or interaction between maternal and fetal HR.

Maternal-fetal HR coupling or interaction was quantified in different literature by using different mathematical methods such as beat-by-beat coupling analysis (15–17), cross-correlation (CC) analysis (18), and transfer entropy (19). The previous literature reported that coupling between both maternal and fetal HR exists and it can be affected by different maternal factors such as sleep (18), respiration (17), and pregnancy weeks (16). Due to discrepancies in the literature concerning the methods that were used to assess coupling or interaction, different conclusions were reported. For example, Leeuwen et al. (17) reported that epochs of synchronization increased with maternal respiratory rate whereas Marzbanrad et al. (19) reported no association between coupling and respiratory rate. The latter conclusions imply that there is still a lack of knowledge regarding the importance of studying maternal-fetal HR coupling in general, moreover, it is unknown which methods may provide more insight into fetal development and wellbeing.

In this study, we aimed at investigating the impact of ASD on maternal-fetal HR coupling or interaction patterns during pregnancy. To achieve our aim, we first investigated how maternal-fetal HR interaction patterns change in mice across different embryonic days (EDs): 13.5, 15.5, 17.5, and 18.5. Fetal mouse development is discussed in our earlier study (20). It was important to study interaction patterns in development to establish an understanding of how maternal-fetal HR interaction change with typical development. After that, we used our previous ASD model (5) to compare between VPA group and saline group (control) to see if maternal-fetal HR interaction patterns change with ASD. To quantify maternal-fetal HR interaction, we investigated maternal and fetal RRI visually to explore the possibility of finding similarities between both. Then, the degree of similarities was quantified by means of CC and magnitude-squared coherence (MSC) analyses.

## Method

### Animal handling

The data used in this study were analyzed retrospectively and they were mentioned in our previous studies (5, 20). Animal handling and experimental protocols were in accordance with the Guidelines for the Care of Laboratory Animals of Tohoku University Graduate School of Medicine. The protocols were approved by the Committee on Animal Experiments at Tohoku University, < city>Sendai</city>, Japan (study approval number: 2017MdA-334). Before mating, C57BL6/J female mice (CLEA, Tokyo, Japan) were housed socially in 3–5 groups in cages under control lightning of 12 h:12 h light-dark cycle. Mice had unlimited access to water and food. For mating, female mice (7–19 weeks of age) were housed in cages with male mice of similar age (1 male and 1 female mouse per cage) in the evening and then separated the next morning. To make the VPA model, 600 mg/kg of valproic acid sodium salt (VPA; Sigma, St. Louis, MO, USA) dissolved in saline solution was injected into the subcutaneous fat of the pregnant mother's neck on ED12.5. The control group of mice had only saline solution injected into them at the same location.

### Experimental protocol

Before collection of electrocardiogram (ECG) records, pregnant mice were anesthetized with subcutaneous administration of ketamine (Ketalar 500 mg, 100 mg/kg; Daiichi-Sankyo, Tokyo, Japan) and xylazine (Rompun 2% w/v solution, 10 mg/kg; Bayer, Leverkusen, Germany) and maintained under anesthetic with inhalational isoflurane (0.5%, 260 ml/min; Forane AbbVie Inc., Chicago, IL, United States). The depth of anesthesia was assessed by using a toe pinch test. The combination of isoflurane with ketamine-xylazine was used to ensure the maintenance of stable anesthesia during ECG measurements. We could successfully use the same anesthetic combinations to measure clear and stable records of fetal ECG (fECG) in our previous studies (5, 21). The ECG recording setup is explained in detail in our previous studies (5, 21, 22). Basically, three needle electrodes were attached to the maternal body to record maternal ECG (mECG). fECG was recorded by inserting two needle electrodes into the uterus to attach them to the chest and back of the fetus. A biomedical amplifier and recording system (Polymate AP1532; TEAC, Tokyo, Japan) was used to record ECG at 1,000 Hz for 15 mins.

#### Fetal mouse development

For fetal mouse development (20), simultaneous records of mECG and fECG of 2 random fetuses (from the same mother) were collected at embryonic days of ED13.5, ED15.5, ED17.5, and ED18.5. The total number of ECG data that were considered for analysis in this study was: ED13.5: 10 mothers (20 fetuses), ED15.5: 8 mothers (16 fetuses), ED17.5: 11 mothers (22 fetuses), ED18.5: 12 mothers (24 fetuses).

#### ASD mouse model

For the ASD mouse model (5), simultaneous records of mECG and fECG of 2 random fetuses (from the same mother) were collected on ED15.5 and ED 18.5. The total number of ECG records that were considered for this study was: E15.5: Saline: 8 mothers (16 fetuses), VPA: 8 mothers (16 fetuses). E18.5: Saline: 8 mothers (16 fetuses), VPA: 7 mothers (14 fetuses).

### Data analysis

All analysis described in this study was conducted in MATLAB 2022a. The first minute of ECG records was excluded from the study due to noise. ECG records were examined to find two segments of 3-minute epochs with no noise or consistent arrhythmia. In all mice, except for 3 cases, the 3-minute epochs were chosen consecutively. The two 3-minute epochs were chosen from the 14 mins of ECG recordings regardless of order (beginning, middle, or end of the recording). Data that did not have at least two 3-minute epochs of clear ECG recordings were excluded from the study. Hence, the total number of data that was included in the analysis is summarized in Table 1. A window size of 3-minute was used for analysis because it was the maximum window size that could be considered with no arrhythmia or noise in the middle in all subjects.

**Table 1:** Summary of sample size.

**Mouse model**	**ED13.5**	**ED15.5**	**ED17.5**	**ED18.5**
Development	6 mothers (10 fetuses)	6 mothers (10 fetuses)	10 mothers (17 fetuses)	7 mothers (11 fetuses)
ASD		**Saline:** 8 mothers (13 fetuses)		**Saline:** 5 mothers (7 fetuses)
		**VPA:** 8 mothers (13 fetuses)		**VPA:** 5 mothers (7 fetuses)

ASD, autism spectrum disorder; ED, embryonic day; VPA, valproic acid.

### Measuring similarities between maternal and fetal RRI tachograms

Maternal and fetal RRI tachograms were resampled at 2 s by taking the average of RRI per 2 s to unify the lengths of both signals. Since ECG signals were collected at a sampling rate of 1 KHz, 3-minute segments would have 180,000 samples. After resampling at 0.5 Hz (2 s or 2,000 samples), the new resampled RRI signals would have 90 samples (180,000/2,000 = 90) per 3-minute segment. A minimum of 2 s was used to accommodate for fetal RRI (fRRI) (the maximum average fRRI was around 928 ms). After that, the resampled signals were normalized by using Equation 1:


(1) $$\begin{array}{l} Normalized\;RRI=\frac{RRI-mean\;RRI}{\max\left(abs\left(RRI-mean\;RRI\right)\right)} \end{array}$$

After normalization, similarities were measured by using “xcorr” (for CC analysis) and “mscohere” (for MSC analysis) functions in MATLAB2022a (23, 24). “xcorr” was used for time-based similarity estimation and “mscohere” was used for frequency-based similarity estimation. To calculate similarity by using “xcorr,” the resampled normalized signal was divided into 10 samples. After that, CC coefficients were calculated with using the “normalized” option in MATLAB per 10 samples. The latter calculation yields a total of 9 CC coefficients per 3 mins (90 samples). To obtain an overall CC score or coefficient for the whole 3-minute epoch, the average of the 9 CC coefficients was calculated. With this approach, two similarity scores were obtained, CC1 and CC2. CC1 was calculated by taking the absolute average whereas CC2 was calculated by taking the average with considering signs.

**Figure 1:**
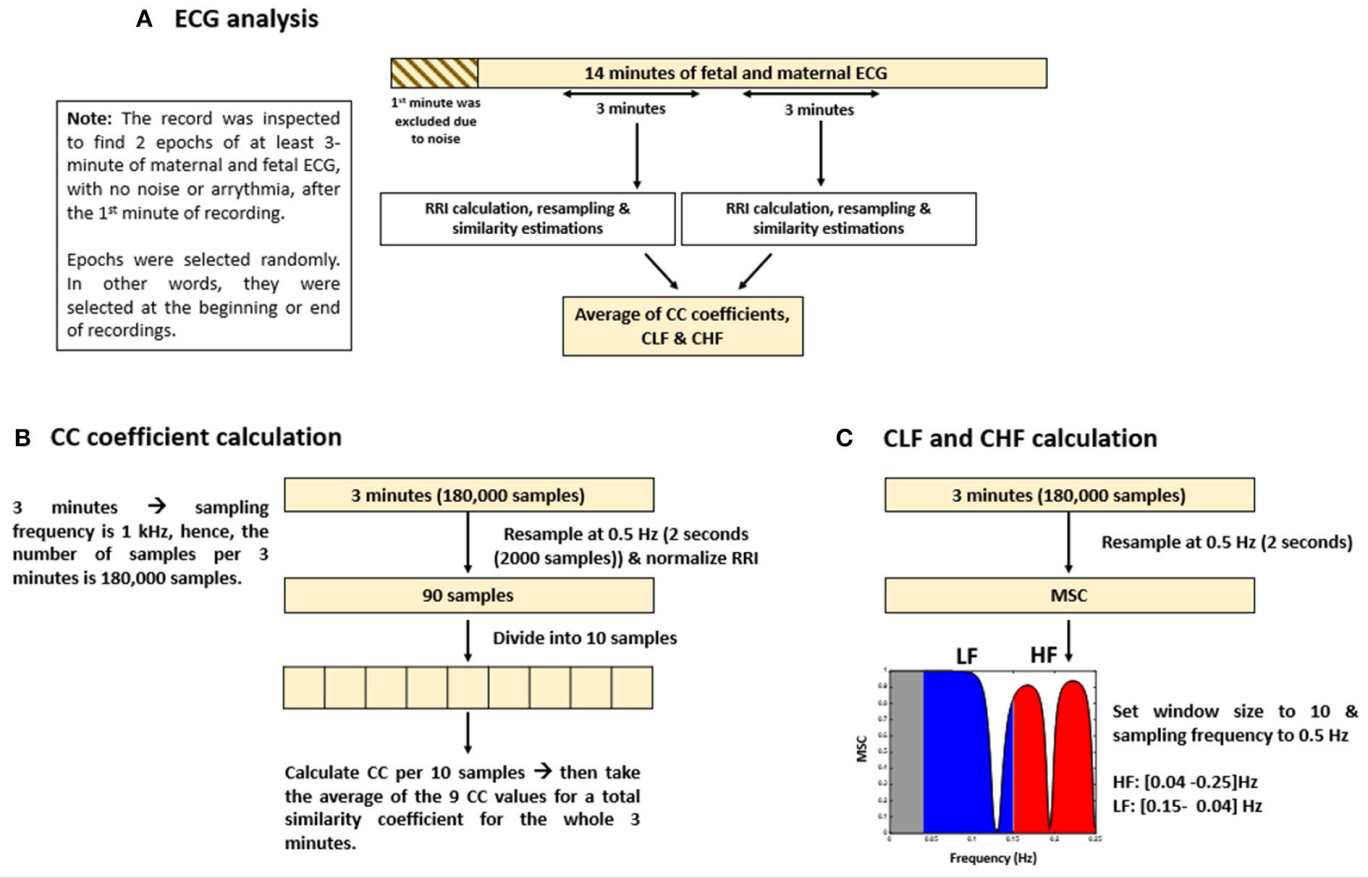
Summary of electrocardiogram (ECG) analysis and similarity estimations. The figure provides **(A)** an illustrative summary of the protocols that were followed to analyze ECG data, **(B)** calculation of cross correlation (CC) coefficients, and **(C)** coherence low frequency (CLF) and coherence high frequency (CHF) calculations.

The methods that were adopted for similarity estimation by using “mscohere” were similar to that of frequency HRV estimation in which the power spectrum is divided into bands. Here, we divided the power spectrum into two bands: low frequency (LF) and high frequency (HF). The maximum frequency obtained from the “mscohere” is 0.25 Hz, so we used the following divisions: LF: (0.04–0.15) Hz, HF: (0.15–0.25) Hz. Since up until now, there are no well-defined bands for fetal mice to measure ANS activity, we used bands defined for humans (25) because the range of average fRRI in our experiment was (306–981) ms. Similarity estimation by “mscohere” was done by setting the window size to 10 and the sampling frequency to 0.5 Hz. After that, the power spectrum density was estimated per band. Similarity for the LF and HF bands will be denoted as coherence LF “CLF” and coherence HF “CHF,” respectively. Figure 1 provides a summary of ECG and similarity analysis.

### Statistical analysis

Before performing statistical analysis, normality tests were conducted in MATLAB2022a by using the Shapiro-Wilk test (swtest) (26). For normally distributed data, correlation coefficients were calculated by Pearson correlation analysis, otherwise, Spearman was used. Comparison of means analysis was performed with one-way ANOVA for normally distributed data and with Wilcoxon test for non-normally distributed data.

## Results

### Fetal mouse development

#### Similarity patterns between maternal and fetal RRI tachograms

**Figure 2:**
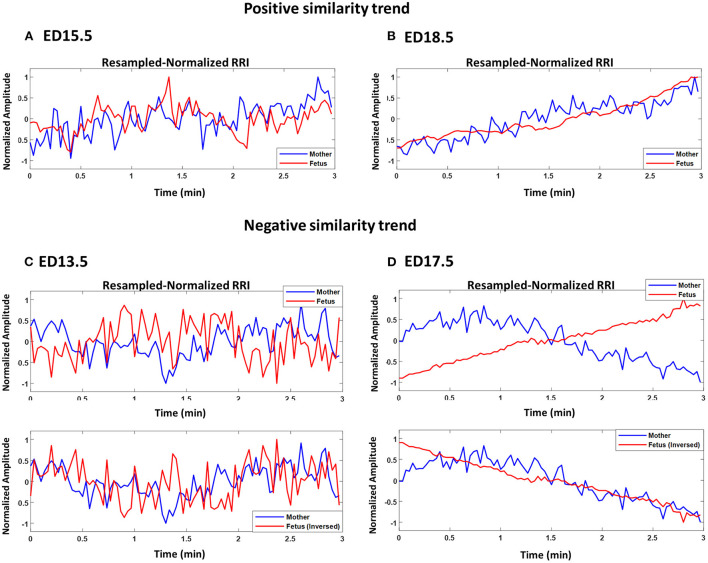
Demonstration of maternal and fetal RR interval (RRI) similarities. All panels show resampled normalized maternal (blue) and fetal (red) RRI. **(A)** ED15.5, CC1: 0.42, CC2: 0.27, coherence low frequency (CLF): 0.084, coherence high frequency (CHF): 0.074. **(B)** ED18.5, CC1: 0.82, CC2: 0.67, CLF: 0.98, CHF: 0.92. **(C)** ED13.5, CC1: 0.29, CC2: −0.18, CLF: 0.086, CHF: 0.074. **(D)** ED17.5, CC1: 0.85, CC2: −0.85, CLF: 0.097, CHF: 0.088.

Figures 2A–D show plots of normalized maternal (blue) and fetal (red) RRI tachograms from different developmental stages. Figures 2A,B show examples of a positive similarity trend whereas Figures 2C,D show examples of a negative similarity trend. In positive similarity trends, maternal and fetal RRI change in the same direction whereas in negative similarity trends, the same change in opposing directions. The upper panels of Figures 2C,D show the original changes in RRI and the lower panels show the same mRRI and the inversed fRRI tachogram to clarify the negative similarity trend. It is noticeable in Figures 2A–D that the degree of similarity between maternal and fetal RRI tachograms is more obvious at late developmental stages (ED17.5 and ED18.5).

**Figure 3:**
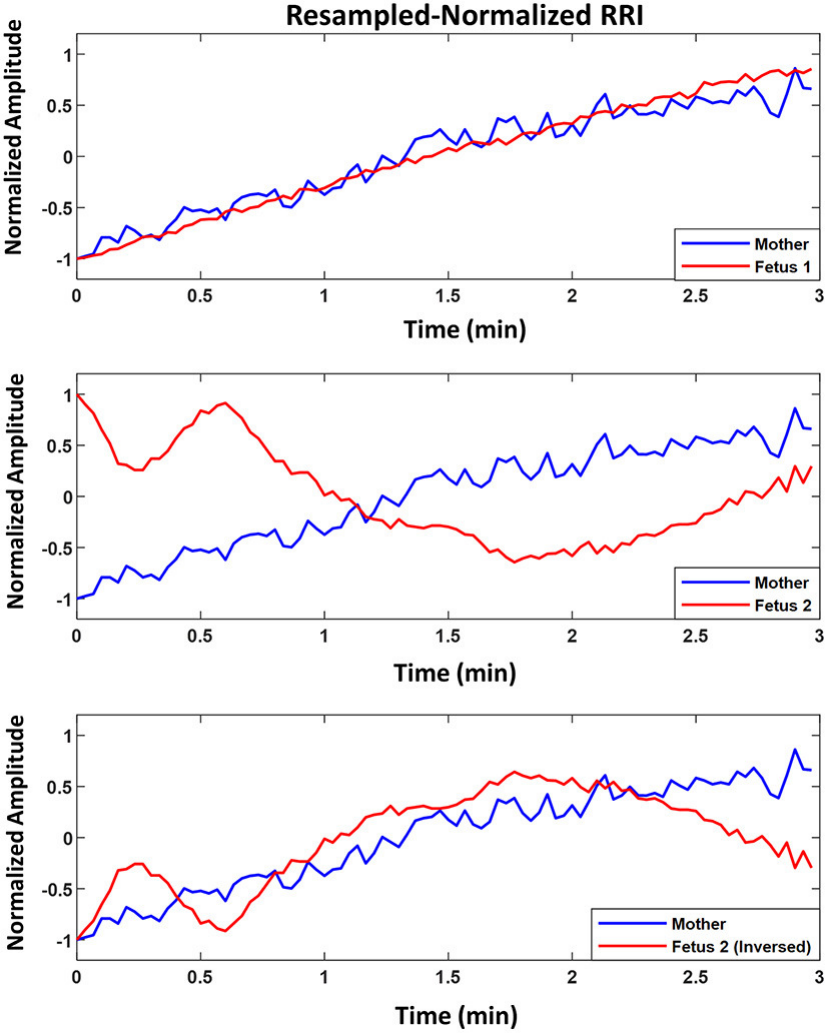
Example of similarity trend of two fetuses from the same mother. The figure shows resampled normalized maternal (blue) and fetal (red) RR interval (RRI) from embryonic day 17.5 (ED17.5). The **Top panel** shows RRI for fetus 1 and the **Middle** and **Bottom panels** show the same for fetus 2. For fetus 1 **(Top panel)**, CC1, CC2, coherence low frequency (CLF) and coherence high frequency (CHF) values were as follows, 0.92, 0.92, 0.098, and 0.093, respectively. For fetus 2, the same latter values were as follows: 0.83, –0.60, 0.098, and 0.092, respectively.

Figure 3 shows an example of two fetuses from ED17.5 which belonged to the same mother with different similarity trends. In all three panels (top, middle, bottom), mRRI is the same. The top panel shows a positive similarity trend for fetus 1. The middle and bottom panels show RRI tachograms that belonged to the second fetus. In the bottom panel, fRRI is inversed to clarify the negative similarity trend. Figure 3 demonstrates that, at a given time, HRs of fetuses that belong to the same mother may correlate differently to maternal HR.

#### The degree of similarity between maternal and fetal RRI increases with fetal age

**Figure 4:**
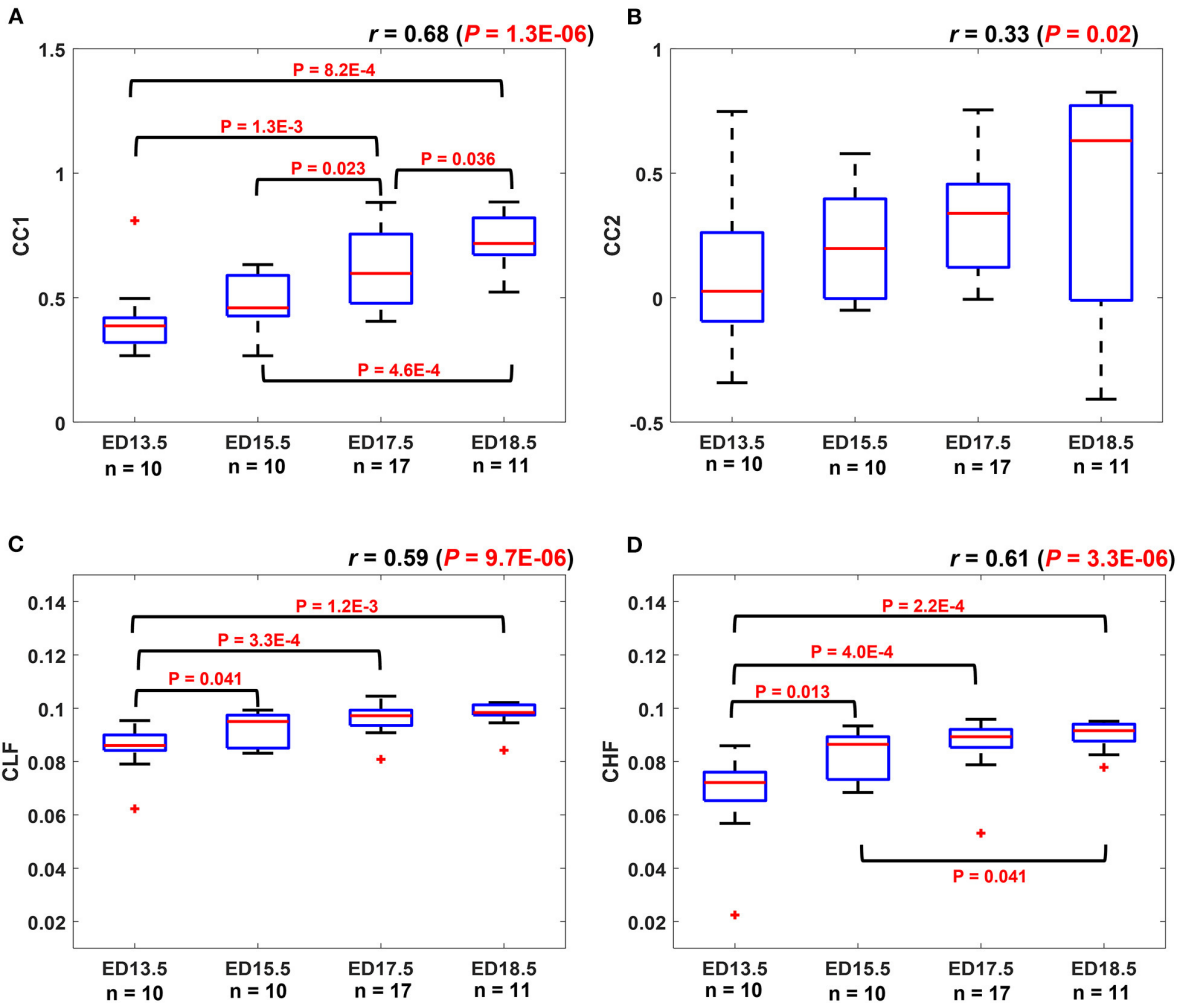
Similarity coefficients at different developmental stages. The similarity between maternal and fetal RR interval (RRI) was quantified by using four coefficients: **(A)** CC1, **(B)** CC2, **(C)** coherence low frequency (CLF), and **(D)** coherence high frequency (CHF). The value of correlation between the coefficient and embryonic days (EDs) is indicated as *r* above the plot.

Figure 4 shows the boxplots for CC1 (A), CC2 (B), CLF (C), and CHF (D) coefficients. In each plot, the value of correlation between the coefficient with EDs is shown as *r*. The average maternal and fetal RRI values for the subjects that were used in Figure 4 are listed in Supplementary Table 1. In all plots, there is an increasing trend in the coefficients indicating an increase in similarity with fetal development. Comparison of means analysis shows that there were no significant differences between any developmental stages in CC2 (Figure 4B), on the other hand, some differences were found in the rest. In Figure 4A, CC1 values at ED17.5 and ED18.5 were significantly higher than in the previous developmental stages. In Figure 4C, CLF values at ED13.5 were significantly lower than ED15.5, ED17.5, and ED18.5. In Figure 4D, CLF values at E18.5 were significantly higher than ED15.5 and ED13.5, also, CLF values at ED17.5 and ED15.5 were significantly higher than ED13.5. The correlation coefficients show positive significant correlations and based on the *r*-value of the CC2 coefficient, it is indicated that the probability of finding positive similarity trends increase with fetal growth.

### ASD mouse model

#### Similarity patterns in VPA-treated mice are disturbed

**Figure 5:**
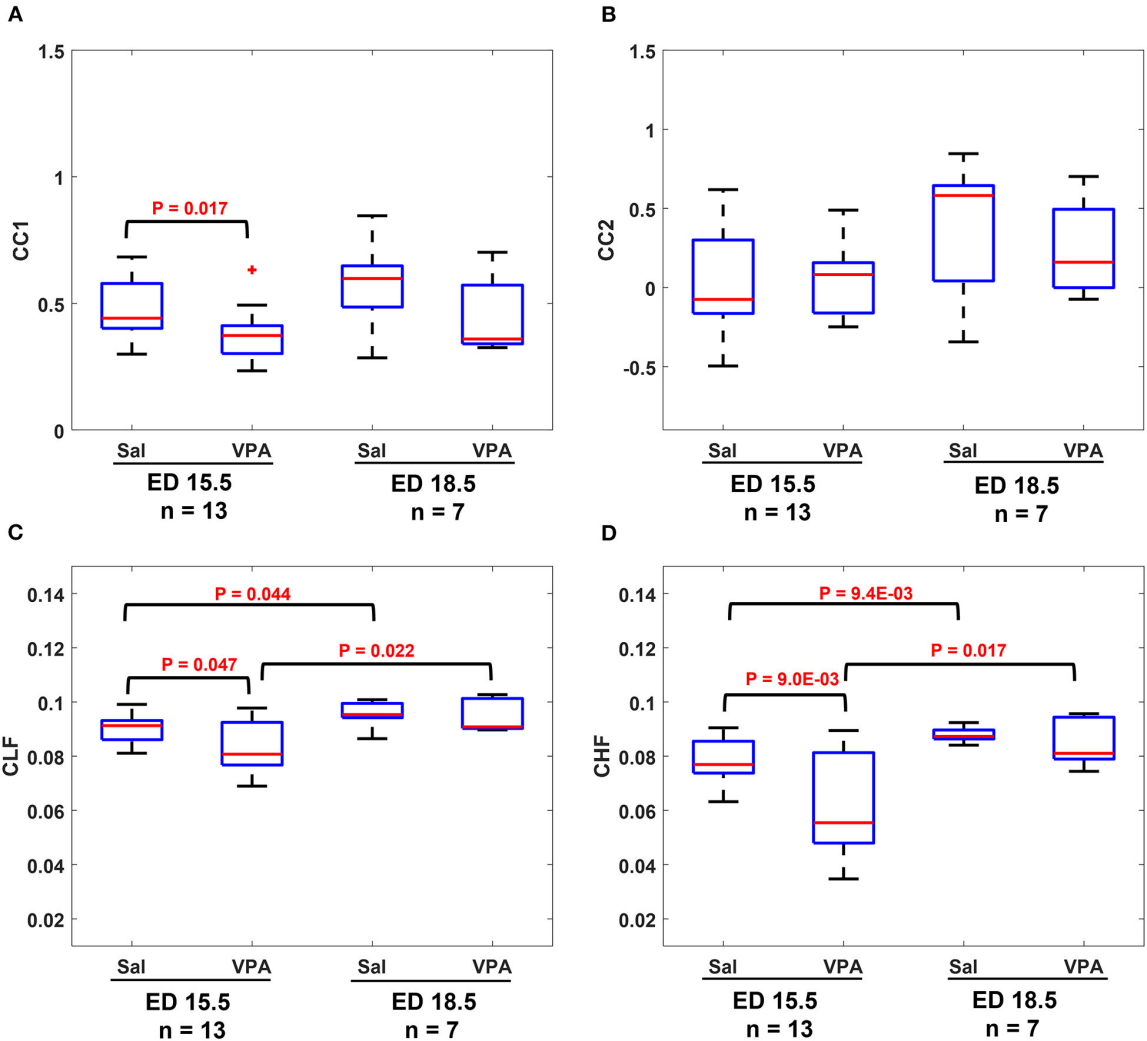
Comparison between saline (sal) group and valproic acid (VPA) groups at embryonic day (ED) 15.5 and ED18.5. **(A)** CC1 coefficient. **(B)** CC2 coefficient. **(C)** Coherence low frequency (CLF) coefficient. **(D)** coherence high frequency (CHF) coefficient.

The results in Figure 4 show that similarities are indicators of fetal development. Hence, we conducted a comparison of means analysis between VPA and saline groups in terms of CC1 (Figure 5A), CC2 (Figure 5B), CLF (Figure 5C), and CHF (Figure 5D). The average maternal and fetal RRI values for the subjects that were used in Figure 5 are listed in Supplementary Table 2. In all figures, except Figure 5B, it is shown that the saline coefficient values at ED15.5 are significantly higher than that of VPA. With respect to the comparison at ED18.5, all figures show that the saline coefficients were found to be higher than that of VPA but the differences were not significant.

## Discussion

We demonstrated that changes exhibited by maternal and fetal RRI share similarities between them in Figures 2A–D, 3. Similarities were found to follow positive trends (Figures 2A,B) or negative trends (Figures 2C,D). The quantity addressed here as similar is the simultaneous rate of change in maternal and fetal RRI. HRV is controlled by ANS and since both maternal and fetal blood circulatory systems and ANS are separated and work independently, the presence of similarities between both RRI suggests the presence of a mediator between both. The mediator functions as a means of communication between maternal and fetal ANS systems. The CLF and CHF results shown in Figures 4C,D indicate the presence of frequency entrainment between the mother and fetus, which suggests that the entrainment of ANS function could be important as a mediator between the mother and fetus. Figure 3 shows that at a particular time, fetuses belonging to the same mother may interact differently with maternal HR and this implies differences in mediator's influences. Since each fetus has an independent placenta, which is the point of connection between the mother and her child, we expect that the effect of the mediator could be modified by placental functions and/or factors such as hormones related to the placenta. Also, because C57BL6/J maternal mice, on average, can give birth to around 5–11 pups (27), we expect the mediation to be affected by the total number of fetuses within a maternal body.

The exact reason behind the similarity is unknown but, according to Figures 4A–D, it seems that the degree of similarity is associated with fetal development. The more advanced the fetal age is, the more fRRI mimics mRRI. Figures 4A,B provide CC results in the time domain and Figures 4C,D provide the same in the frequency domain. According to Figures 4A,B, the *r* value in CC2 (Figure 4B) was lower than that of CC1 (Figure 4A), in addition, the comparison of means analysis showed that there were no differences between any of the developmental stages in CC2. This implies that CC1 is more indicative of fetal development compared to CC2. Since CC1, which was evaluated by absolute value, showed a better association with fetal development than CC2, it is implied that a typical development needs to have an increased correlation between a mother and her fetus, whether positive or negative. Positive and negative correlations between mother and fetus may have some significance, but we cannot elaborate more about them in this study, and we believe that they need to be verified by other approaches. With respect to frequency-based CC analysis, it is revealed that both CLF and CHF were indicative of fetal development.

Previously (20), we used HRV analysis to assess fetal ANS development in which we considered the LF band as an indicator of the sympathetic and parasympathetic systems activity and the HF band as an indicator of the parasympathetic system activity. We do not know how our CLF and CHF associate exactly with either maternal or fetal sympathetic or parasympathetic systems, but we speculate that they associate with ANS activity in general. Mathematically, MSC measures how two signals correlate or are similar in the frequency domain (28), and in our study, the two signals consisted of maternal and fetal RRI, therefore, CLF and CHF are combined measures of maternal and fetal HF and LF. In our study, the CHF factor was more efficient compared to CLF in terms of distinguishing between developmental stages. In Figure 4D, there was a significant difference between ED15.5 and ED18.5 whereas the same was absent in Figure 4C. The latter pattern was observed in our earlier study (20) in which we found that the fetal HF band was significantly more correlated to EDs compared to LF.

The results from fetal development highlight this fact, an association between maternal and fetal RRI or ANS is a feature of development and disturbances in such correlation are expected to impair fetal development. In the ASD mouse model (Figures 5A–D), it is revealed that there were significant differences between control and VPA groups in CC1, CLF, and CHF values at ED15.5. As in the case of fetal development, CC1 was found to be more appropriate than CC2 in this study, because we could not detect any differences due to VPA administration by CC2. At ED18.5, all four values in the VPA group were found to be generally lower but with no significance. The absence of significance implies that VPA mice could eventually achieve development, but compared to the control group, their development was delayed, and this could be a feature of ASD that is observed only in the fetal period.

The main conclusion that can be highlighted from Figures 5A–D is disturbances in communication between maternal and fetal ANS or HRV is a feature of ASD. Intriguingly, such disturbances were found to be manifested in ED15.5 only which suggests that impairment in fetal neural development could be masked with fetal age and growth and the effect of such impairment seems to take effect after childbirth and growth. The findings in Figures 5A–D imply that the miscommunication that started between maternal and fetal HR in the intrauterine environment gets manifested as miscommunication between the child and his/her surroundings after birth. These results are consistent with the developmental origin of health and disease (DOHaD) and fetal programming theories which associate the origin of adulthood disorders with the prenatal period (29–31).

Comparison between Figures 4, 5 show that CC2 and CHF trends were consistent in both figures. On the other hand, CC1 and CLF trends were different between both figures in terms of the difference between ED15.5 and ED18.5. In Figures 4A, 5C, the differences were significant whereas they were not significant in Figures 4C, 5A. Although it is not shown in Figure 4C, the *p*-value for the difference between ED15.5 and ED18.5 was 0.053 which is close to the significance level. The absence of significance between ED15.5 and ED18.5 in Figure 5A could be attributed to two reasons. The first is the lower sample size at ED18.5 (ASD mouse model, Figure 5) and the second reason could be attributed to the anesthetic effect. As was demonstrated in the Methods section, two segments of 3-minute were selected randomly from the ECG record for analysis, and such selections were made at the beginning or end of the recordings. Since anesthesia is known to affect HRs in mice (32, 33), the effect of anesthesia is expected to change along the recording. For example, the analysis of HR at the beginning of the recordings could be different from the end of the recordings. The effect of anesthesia is a limitation in our study.

There are several limitations to our study. MSC calculation requires signals to have the same lengths, therefore we resampled our signals to unify lengths. As a result of resampling, information regarding CHF was partially lost. CC analysis was used to calculate CC2 to assess directionality in the similarity between maternal and fetal RRI. In Figure 4, the *r* value was low in Figure 4B, also, there were no significant differences between any of the developmental stages in Figure 4B. In Figure 5B, the CC2 value was not effective in distinguishing between VPA and saline. Hence, we think that more research is needed to effectively estimate the directionality in maternal-fetal RRI similarity.

Here, we analyzed the data retrospectively and the anesthesia mixture (ketamine/xylazine/isoflurane) that was used during ECG recordings is known to affect HR in mice (32, 33). In humans, fetal HR and HRV are known to be affected by fetal gender and behavioral states (10, 34) and we expect the same effect to exist in mice. Therefore, we speculate that fetal gender, behavioral states and anesthesia affected CC, CLF and CHF values in our study. Due to our retrospective design, we did not have further information regarding fetal gender. The lower number of subjects of saline and VPA at ED18.5 was another limitation.

## Conclusion

Our study highlighted the presence of similarities between maternal and fetal RRI tachograms in mice. We quantified the similarities by using four correlation coefficients and we found that the degree of similarity increased with fetal age in typical fetal mouse development. We further investigated the similarity patterns in the ASD mouse model treated with VPA. Comparison of means analysis between VPA and saline mice showed that the correlation coefficients were generally reduced in VPA mice indicating a disturbance in the rhythm or regulation by which fetal and maternal HRs interact. Hence, we conclude that impairment in fetal and maternal HRs interaction could be a possible feature of ASD during pregnancy.

## Data availability statement

The original contributions presented in the study are included in the article/Supplementary material, further inquiries can be directed to YKa: kasa@med.tohoku.ac.jp.

## Ethics statement

The animal study was reviewed and approved by the Committee on Animal Experiments in Tohoku University.

## Author contributions

NW performed research, analyzed data, and wrote the paper with inputs from all authors. AK, YKi, and MS supervised the study and provided resources and funding. CY, KN, MF, and AS collected and organized data and performed experiment. Yka contributed to conception and design of the study, supervised the study, collected data, and provided resources and funding. All authors contributed to manuscript revision, read, and approved the submitted version.

## Funding

The work in this paper has been supported by RIKEN Healthcare and Medical Data Platform Project, the funding for Basic Medical Research by Shiguredo Inc. and collaborative CIRA grant (2019-023) awarded to AK by Khalifa University Abu Dhabi. Also, the research is partially supported by the Project for Baby and Infant in Research of Health and Development to Adolescent and Young adult from Japan Agency for Medical Research and development, AMED.

## Conflict of interest

This study received funding from Shiguredo Inc. The funder was not involved in the study design, collection, analysis, interpretation of data, the writing of this article, and the decision to submit it for publication.

The authors declare that the research was conducted in the absence of any commercial or financial relationships that could be construed as a potential conflict of interest.

## Publisher's note

All claims expressed in this article are solely those of the authors and do not necessarily represent those of their affiliated organizations, or those of the publisher, the editors and the reviewers. Any product that may be evaluated in this article, or claim that may be made by its manufacturer, is not guaranteed or endorsed by the publisher.
