# Supplementary material for: Correlation between maternal and fetal heart rate increases with fetal mouse age in typical development and is disturbed in autism mouse model treated with valproic acid
Source: Front Psychiatry. 2022 Nov 24;13:998695. doi: 10.3389/fpsyt.2022.998695 (PMC9743798; doi:10.3389/fpsyt.2022.998695)
Supplement: Supplementary file 1 [file Data_Sheet_1.docx]

**Correlation between maternal and fetal heart rate increases with fetal mouse age in typical development and is disturbed in autism mouse model treated with valproic acid**

Namareq Widatalla, Ahsan Khandoker, Chihiro Yoshida, Kana Nakanishi, Miyabi Fukase, Arisa Suzuki, Masatoshi Saito, Yoshitaka Kimura, Yoshiyuki Kasahara

**Supplementary material**

**Developmental data set**

The table shows average values of maternal and fetal RR interval (RRI) along with the standard deviation (std) from the developmental data set. The first column indicates the maternal subject number. In case data from two fetuses were collected per one mother, two rows are assigned to that mother to indicate the average RRI per fetus.

**Supplementary Table 1: Developmental data set**

| **Subject Number** | **Maternal RRI (mean ± std)** | **Fetal RRI (mean ± std)** |
| --- | --- | --- |
| **ED* 13.5** | | |
| **Mother 1** | 234 ± 10 | 611 ± 7.6 |
|  |  | 536 ± 6.3 |
| **Mother 2** | 215 ± 3.2 | 739 ± 14 |
| **Mother 3** | 244 ± 7.3 | 749 ± 8.6 |
| **Mother 4** | 195 ± 4.2 | 936 ± 41 |
| **Mother 5** | 245 ± 17 | 777 ± 54 |
| **Mother 6** | 268 ± 11 | 628 ± 10.6 |
|  |  | 530 ± 4.3 |
| **Mother 7** | 205 ± 2.7 | 673 ± 20 |
|  |  | 686 ± 17 |
| **ED 15.5** | | |
| **Mother 1** | 255 ± 2.2 | 843 ± 14 |
|  |  | 843 ± 16 |
| **Mother 2** | 256 ± 1.3 | 963 ± 23 |
| **Mother 3** | 214 ± 5.1 | 678 ± 8.1 |
|  |  | 564 ± 19 |
| **Mother 4** | 338 ± 16 | 574 ± 13 |
|  |  | 589 ± 35 |
| **Mother 5** | 219 ± 5.9 | 645 ± 11 |
|  |  | 470 ± 1.5 |
| **Mother 6** | 182 ± 2.3 | 543 ± 16 |
| **ED 17.5** | | |
| **Mother 1** | 240 ± 7.2 | 817 ± 7.3 |
|  |  | 803 ± 8.6 |
| **Mother 2** | 228 ± 5.4 | 691 ± 19 |
|  |  | 884 ± 36 |
| **Mother 3** | 304 ± 9.6 | 798 ± 14 |
| **Mother 4** | 304 ± 5.4 | 891 ± 4.1 |
| **Mother 5** | 178 ± 3.4 | 700 ± 22 |
|  |  | 529 ± 3.7 |
| **Mother 6** | 200 ± 3.3 | 649 ± 9.6 |
|  |  | 655 ± 6.6 |
| **Mother 7** | 179 ± 1.3 | 742 ± 26 |
|  |  | 550 ± 8.9 |
| **Mother 8** | 186 ± 3.9 | 649 ± 32 |
| **Mother 9** | 202 ± 7.1 | 570 ± 14 |
|  |  | 571 ± 16 |
| **Mother 10** | 222 ± 3.3 | 687 ± 12 |
|  |  | 619 ± 3.9 |
| **ED 18.5** | | |
| **Mother 1** | 221 ± 4.2 | 524 ± 6.1 |
|  |  | 634 ± 28 |
| **Mother 2** | 208 ± 4.8 | 428 ± 7.6 |
|  |  | 626 ± 19 |
| **Mother 3** | 211 ± 2.9 | 573 ± 27 |
| **Mother 4** | 238 ± 3.9 | 601 ± 8.5 |
| **Mother 5** | 180 ± 1.8 | 515 ± 14 |
|  |  | 394 ± 16 |
| **Mother 6** | 169 ± 2.5 | 540 ± 16 |
|  |  | 414 ± 16 |
| **Mother 7** | 172 ± 2.5 | 569 ± 64 |

**ED: embryonic day, RRI: RR interval.**

**Autism model data set**

The table shows average values of maternal and fetal RRI along with the std from the autism data set. The first column indicates the maternal subject number. In case data from two fetuses were collected per one mother, two rows are assigned to that mother to indicate the average RRI per fetus.

**Supplementary Table 2: Saline E15.5**

| **Subject Number** | **Maternal RRI (mean ± std)** | **Fetal RRI (mean ± std)** |
| --- | --- | --- |
| **Saline ED 15.5** | | |
| **Mother 1** | 213 ± 3.9 | 503 ± 3.25 |
| **Mother 2** | 274 ± 5.6 | 818 ± 14 |
| **Mother 3** | 275 ± 11 | 846 ± 13 |
|  |  | 835 ± 6.7 |
| **Mother 4** | 240 ± 4.07 | 685 ± 6.8 |
|  |  | 690 ± 29 |
| **Mother 5** | 216 ± 5.6 | 608 ± 21 |
|  |  | 767 ± 28 |
| **Mother 6** | 265 ± 9.6 | 885 ± 13 |
|  |  | 863 ± 19 |
| **Mother 7** | 235 ± 4.9 | 709 ± 6.0 |
| **Mother 8** | 229 ± 10 | 533 ± 17 |
|  |  | 794 ± 50 |
| **VPA ED 15.5** | | |
| **Mother 1** | 256 ± 2.3 | 788 ± 6.1 |
|  |  | 819 ± 26 |
| **Mother 2** | 267 ± 14 | 741 ± 15 |
|  |  | 725 ± 17 |
| **Mother 3** | 271 ± 4.9 | 838 ± 11 |
|  |  | 809 ± 23 |
| **Mother 4** | 254 ± 2.7 | 854 ± 30 |
|  |  | 845 ± 5.4 |
| **Mother 5** | 271 ± 10 | 557 ± 37 |
| **Mother 6** | 282 ± 9.1 | 755 ± 36 |
|  |  | 748 ± 14 |
| **Mother 7** | 258 ± 6.1 | 723 ± 41 |
| **Mother 8** | 255 ± 6.5 | 934 ± 32 |
| **Saline ED18.5** | | |
| **Mother 1** | 213 ± 2.5 | 453 ± 2.6 |
| **Mother 2** | 212 ± 3.4 | 557 ± 33 |
| **Mother 3** | 272 ± 6.3 | 318 ± 4.9 |
|  |  | 353 ± 9.1 |
| **Mother 4** | 233 ± 7.8 | 395 ± 6.0 |
|  |  | 367 ± 4.7 |
| **Mother 5** | 213 ± 5.5 | 331 ± 14 |
| **VPA ED18.5** | | |
| **Mother 1** | 207 ± 1.4 | 476 ± 5.1 |
|  |  | 451 ± 1.7 |
| **Mother 2** | 260 ± 9.1 | 448 ± 2.1 |
|  |  | 382 ± 4.6 |
| **Mother 3** | 251 ± 11 | 416 ± 2.5 |
| **Mother 4** | 210 ± 3.9 | 478 ± 7.0 |
| **Mother 5** | 272 ± 8.3 | 549 ± 4.8 |

**ED: embryonic day, RRI: RR interval, VPA: valproic acid.**
